# Establishment of a patient-derived intrahepatic cholangiocarcinoma xenograft model with KRAS mutation

**DOI:** 10.1186/s12885-016-2136-1

**Published:** 2016-02-11

**Authors:** Giuliana Cavalloni, Caterina Peraldo-Neia, Francesco Sassi, Giovanna Chiorino, Ivana Sarotto, Massimo Aglietta, Francesco Leone

**Affiliations:** Fondazione del Piemonte per l’Oncologia (FPO), Candiolo Cancer Institute-IRCCS, Candiolo, Italy; Unit of Molecular Pharmacology, Candiolo Cancer Institute-IRCCS, University of Turin Medical School, Candiolo, Italy; Cancer Genomics Laboratory, Fondazione Edo ed Elvo Tempia Valenta, Biella, Italy; Fondazione del Piemonte per l’Oncologia (FPO), Unit of Pathology, Candiolo Cancer Institute-IRCCS, Candiolo, Italy; Oncology Department, Candiolo Cancer Institute-IRCCS, University of Turin Medical School, Candiolo, Italy

**Keywords:** Intrahepatic cholangiocarcinoma, Patient derived xenograft, K-RAS mutation

## Abstract

**Background:**

Intrahepatic cholangiocarcinoma (ICC) is an aggressive, highly lethal tumors and lacks of effective chemo and targeted therapies. Cell lines and animal models, even partially reflecting tumor characteristics, have limits to study ICC biology and drug response. In this work, we created and characterized a novel ICC patient-derived xenograft (PDX) model of Italian origin.

**Methods:**

Seventeen primary ICC tumors derived from Italian patients were implanted into NOD (Non-Obese Diabetic)/Shi-SCID (severe combined immunodeficient) mice. To verify if the original tumor characteristics were maintained in PDX, immunohistochemical (cytokeratin 7, 17, 19, and epithelial membrane antigen) molecular (gene and microRNA expression profiling) and genetic analyses (comparative genomic hybridization array, and mutational analysis of the kinase domain of EGFR coding sequence, from exons 18 to 21, exons 2 to 4 of K-RAS, exons 2 to 4 of N-RAS, exons 9 and 20 of PI3KCA, and exon 15 of B-RAF) were performed after tumor stabilization.

**Results:**

One out of 17 (5.8 %) tumors successfully engrafted in mice. A high molecular and genetic concordance between primary tumor (PR) and PDX was confirmed by the evaluation of biliary epithelial markers, tissue architecture, genetic aberrations (including K-RAS G12D mutation), and transcriptomic and microRNA profiles.

**Conclusions:**

For the first time, we established a new ICC PDX model which reflects the histology and genetic characteristics of the primary tumor; this model could represent a valuable tool to understand the tumor biology and the progression of ICC as well as to develop novel therapies for ICC patients.

**Electronic supplementary material:**

The online version of this article (doi:10.1186/s12885-016-2136-1) contains supplementary material, which is available to authorized users.

## Background

Cholangiocarcinoma (CCA) is the most common biliary tract neoplasm of the biliary tree, classified, according to its site of origin, as intrahepatic (ICC), perihilar or extrahepatic (ECC) cholangiocarcinoma [1, 2]. These subtypes differ in their biology, clinical-pathological characteristics and management. ICC accounts for approximately 10-15 % of CCA [3, 4], although its incidence is different worldwide with a higher incidence in Asia (96 per 100,000 in Thailand) [5], but is increasing also in other geographic regions [6]. Several risk factors of CCA, include infectious and inflammatory diseases, congenital conditions, drugs, and toxins. However, recent studies identified new and emerging risk factors for ICC, occupational and environment-related [7, 8]: the chronic viral hepatitis, liver cirrhosis-alcohol-related, smoking, obesity, diabetes and asbestos [9–13]. Patients with unresectable disease (70-90 %) have a poor prognosis with a survival of less than 12 months following diagnosis.

The lack of effective therapies prompts to identify alternative approaches, based on a deepen molecular knowledge. The high throughput techniques, i.e. gene and microRNA profiling, next generation sequencing (NGS), exome sequencing, provide huge amount of data and information suitable to identify potential drug targets [14, 15]. Nowadays, pathogenesis and drug response are usually studied on preclinical models represented by cell lines, primary cultures, and xenografts.

In particular, xenografts and orthotopic models obtained by CCA cell lines, carcinogen-induced and genetically engineered mouse model for CCA has been created [16]. In the last years, patient-derived cancer xenograft (PDX) models have been established by directly engrafting surgically resected human tumor tissues into immune compromised mice. Molecular and genetic analysis demonstrated that PDXs rely primary tumor characteristics, making them suitable models to study pathogenesis and to test anti-cancer drugs activity. PDXs are established from different cancer types, including gastric, breast, ovarian, colon, lung, prostate, and pancreatic cancers [17–23].

To date, no human CCA models derived from tumor patients have been developed.

Here, we established and characterized a patient-derived ICC model derived from a patient of Italian origin. This model will be helpful either to provide a more suitable model for preclinical studies or to test drug efficacy.

## Methods

### Establishment and characterization of patient derived xenograft (PDX)

Tumor samples were obtained from Italian patients subjected to surgical resection for ICC. Biological material was obtained from patients who has signed the informed consent, following institutional review board-approved protocols (“PROFILING Protocol, n° 001-IRCC-00 IIS-10” approved by Comitato Etico Interaziendale of A.O.U. San Luigi Gonzaga, Orbassano, Torino, Italy). This institutional study provides molecular genetic analysis, set up of primary cultures and the creation of PDX from tumor biological samples (primary tumor, metastasis, tumor cells taken under paracentesis or thoracentesis procedures, and blood). We have overall implanted 17 fresh tumor specimens from ICC patients, 14 primary (PR) and 3 recurrent tumors, here named from CHC001 to CHC020.

For PDX establishment, NOD (Non-Obese Diabetic)/Shi-SCID (severe combined immunodeficient) female mice (4–6 weeks old) (Charles River Laboratory) were maintained under sterile conditions in micro-isolator cages at the animal facilities of IRCCS-Candiolo. All animal procedures were approved by the Institutional Ethical Commission for Animal Experimentation (Fondazione Piemontese per la Ricerca sul Cancro) and by the Italian Ministry of Health. Mice were subcutaneously grafted with a fragment of 4x4 mm of representative tumor.

### Immunohistochemistry analysis

The expression of biliary markers Cytokeratin (CK) 7, 17, 19, and epithelial membrane antigen (EMA) [24] was evaluated by immunohistochemical analysis (IHC) to compare the characteristics of primary and engrafted tumor. Slides were incubated with primary antibodies followed by the appropriate secondary antibodies; the reaction was visualized by DAB (3,3-diaminiobenzidine) and counterstained with hematoxylin.

### Comparative genomic hybridization array

Genomic DNA of PR and its PDX at fourth generation was extracted from formalin fixed, paraffin embedded (FFPE) tissues using the QiAmp FFPE DNA mini Kit (Qiagen). High-resolution oligonucleotide comparative genomic hybridization (CGH) arrays analysis was performed following standard operating procedures of Agilent Technologies. One thousand ng of DNA were digested by a double enzymatic digestion (AluIþRsa I), fragmented, amplified, and purified. After the quantification with Nanodrop, 2 μg of genomic DNA of both tumor and control from Promega (Human Genomic DNA Female N 30742202/male N 30993901) were labeled with CY5-dCTPs and CY3-dCTP, respectively, and hybridized on glass arrays (2 X105 K) at 65C° for 40 hours at 20 rpm. Slides were then washed, scanned on an Agilent 4000C dual laser scanner and images analyzed with Feature Extraction v10.5 software. Raw txt files were then loaded into Cytogenomics software for data processing and visualization.

### Gene and microRNA expression analysis

For gene expression analysis (GEP), tissues were homogenized by using TissueLyser LT (Qiagen s.r.l. Milano, Italy) and total RNA (mRNA and microRNA) was extracted and purified by Absolutely RNA miRNA kit (Agilent Technologies), following manufacturers’ protocols. Quantitative and qualitative evaluation of total RNA was performed by Nanodrop and BioAnalyzer, respectively. For GEP analysis, 100 ng of total RNA were amplified and labeled using Low Input Quick Amp Labeling Kit, one-color kit (Agilent Technologies). Six hundred ng of labeled RNA were hybridized on SurePrint G3 Human Gene Expression 8x60K v2 glass arrays. Arrays were scanned and images analyzed by the Feature Extraction Software from Agilent Technologies (version 10.7); raw data were then processed using the Bioconductor package Limma (Linear models for microarray analysis). Background correction was performed with the *normexp* method with an offset of 50, and *quantile* was used for the between-array normalization. The empirical Bayes method was used to compute a moderated t-statistics.

For microRNA analysis, 100 ng of total RNA were labeled using the miRNA Complete Labeling and Hyb Kit and hybridized on Human miRNA Microarray Kit Release 16.0, 8x60K. Arrays were scanned and images analyzed by the Feature Extraction Software from Agilent Technologies (version 10.7). Raw data elaboration was carried out with Bioconductor (http://www.bioconductor.org/) [25], using R statistical language. Background correction was performed with the normexp method, and quantile was used for the between-array normalization. External datasets: GSE26566 and GSE47764 datasets, containing normal bile duct gene and miRNA expression profiles respectively, were downloaded from the GEO website (http://www.ncbi.nlm.nih.gov/geo/). To merge these raw data to our own, we first averaged the signal at probe level (for microRNA arrays, performed on two different versions of Agilent platform) or at gene symbol level (for gene expression arrays, performed on two different platforms). The obtained matrices were then merged and normalized with the quantile function. The LIMMA (LInear Models for Microarray Analysis) package was used to identify differentially expressed genes/microRNAs in tumor versus normal samples. The empirical Bayes method was used to compute a moderated t-statistics [26].

### MicroRNA validation by qRT-PCR

MicroRNA of PDX and of a pool of liver normal tissues was transcribed in cDNA by using TaqMan microRNA Reverse Transcription Kit (Applied Biosystem) using specific primers for mir-21, mir-199, mir-200, mir-31, and for the housekeeping RPL-21. The TaqMan microRNA Assays (with the different fluorescent probes) and the TaqMan Universal MasterMix NO Amperase UNG were used to perform the quantitative Real-time PCR. All the experiments were carried out in triplicate in optical grade 96-well plates. Quantitative analysis was performed by the measurement of Ct values; briefly, to calculate the relative expression of the target microRNA normalized to RPL21, the average of target C_t_ was subtracted from the average of RPL21 C_t_(ΔC_t_). The amount of target, normalized to an endogenous reference and relative to a calibrator (fold-change) is given by 2^-ΔΔCt^ where the calculation of ΔΔCt involves subtraction by the ΔCt calibrator value (pool of liver normal tissues).

### Mutational analysis

Genomic DNA was extracted by using QIAamp DNA FFPE Mini kit (Qiagen, Milan, Italy) following the manufactures’s instructions. For formalin fixed and paraffin embedded (FFPE) tumor the neoplastic area was obtained by laser microdissection (VSL-337ND-S, Spectra-Physics, Mountain View, CA). The kinase domain of EGFR coding sequence, from exons 18 to 21, was amplified by using primers and nested polymerase chain reaction (PCR) conditions previously described by Lynch and coll [27]. Exons 2 to 4 of K-RAS and N-RAS, exons 9 and 20 of PI3KCA, exon 15 of B-RAF were amplified by PCR as previously described [28, 29]. PCR products were then purified using Wizard® SV Gel and PCR Clean-Up System (Promega, Italy) and sense and antisense sequences were obtained using forward and reverse internal primers, respectively. Each exon was sequenced using the BigDye Terminator Cycle sequence following the PE Applied Biosystem strategy and Applied Biosystem ABI PRISM3100 DNA Sequencer (Applied Biosystem, Forster City, CA). Mutations were confirmed performing two independent PCR amplifications.

## Results

### Generation and characterization of BTC patient derived xenografts

ICC tumors obtained from surgery were subcutaneously implanted into NOD/SCID mice as described in the Materials and Methods section. Characteristics of tumor patients were summarized in Table 1. Ten patients were females and seven males and the age ranged from 44 to 82; 14 out of 17 (82.4 %) tumor specimens were primary tumors and 3 out of 17 (17.6 %) were recurrences.

**Table 1:** Clinical-pathological characteristics of ICC patients

Tumor	Primary/Recurrence	HBV-HCV	TNM	Tumor size	K-RAS mutation	Smoking status
CHC-001	primary	neg	p T2b pN0 G2	40 mm	KRAS G12A	Yes
CHC-002	recurrence	HBV pos	r pT2b N0 G3	27 mm	WT	No
CHC-003	primary	neg	p T2 N1 G2	35 mm	WT	No
CHC-005	primary	neg	pT2b pN0 G3	90 mm	WT	Yes
CHC-006	primary	neg	pT2N0 G3	NA	WT	No
CHC-007	primary	neg	pT4 pN0 G3	70 mm	WT	No
CHC-009	primary	neg	pT2b pN0G2-G3	65 mm	WT	No
CHC-010	primary	HCV pos	pT2a pN0 G3	80 mm	WT	No
CHC-011	primary	neg	pT2b pN0 G3	45 mm	WT	Yes
CHC012	recurrence	neg	r pT2b G2	45 mm	WT	No
CHC013	primary	HBV pos	pT2a G3	NA	WT	No
CHC014	primary	neg	pT3 N1 G3	115 mm	WT	No
CHC015	primary	neg	p T2a pN1 G3	100 mm	WT	No
CHC017	recurrence	neg	r pT2a pN0 G2	25 mm	WT	No
CHC018	primary	neg	pT3 N1 G3	75 mm	WT	Yes
CHC019	primary	neg	pT2bN1 G3	15 mm	WT	No
CHC020	primary	neg	pT2b pN0 G2	110 mm	WT	No

*F* Female, *M* Male, *Neg* negative, *Pos* positive, *NA* not available

Only one tumor out of 17 (5.8 %) was successfully engrafted. It was a primary tumor and was histopathologically classified as pT2b pN0, moderately differentiated (G2) ICC. Tumor sample was also evaluated for the presence of HBV or HCV markers, resulting negative. Patient had chronic colecystitis, but did not have liver cirrhosis or chronic liver disease, primary sclerosing cholangitis diabetes, obesity.

Primary tumor, named CHC001 PDX, was successfully engrafted in mice at the first generation after 4 months; after reaching a volume of 1000 mm^3^, tumor was explanted and re-implanted in new mice. Starting from the second generation, the latency of growth was decreased from 4 months to 1 month until the stabilization obtained at the fourth generation. If cryopreserved in DMSO 10 % and FBS 90 % in culture medium, it was able to successfully engraft in mice when re-implanted. After stabilization, immunohistochemical and molecular investigations were performed to verify if both features were retained in the PDX.

**Fig. 1:**
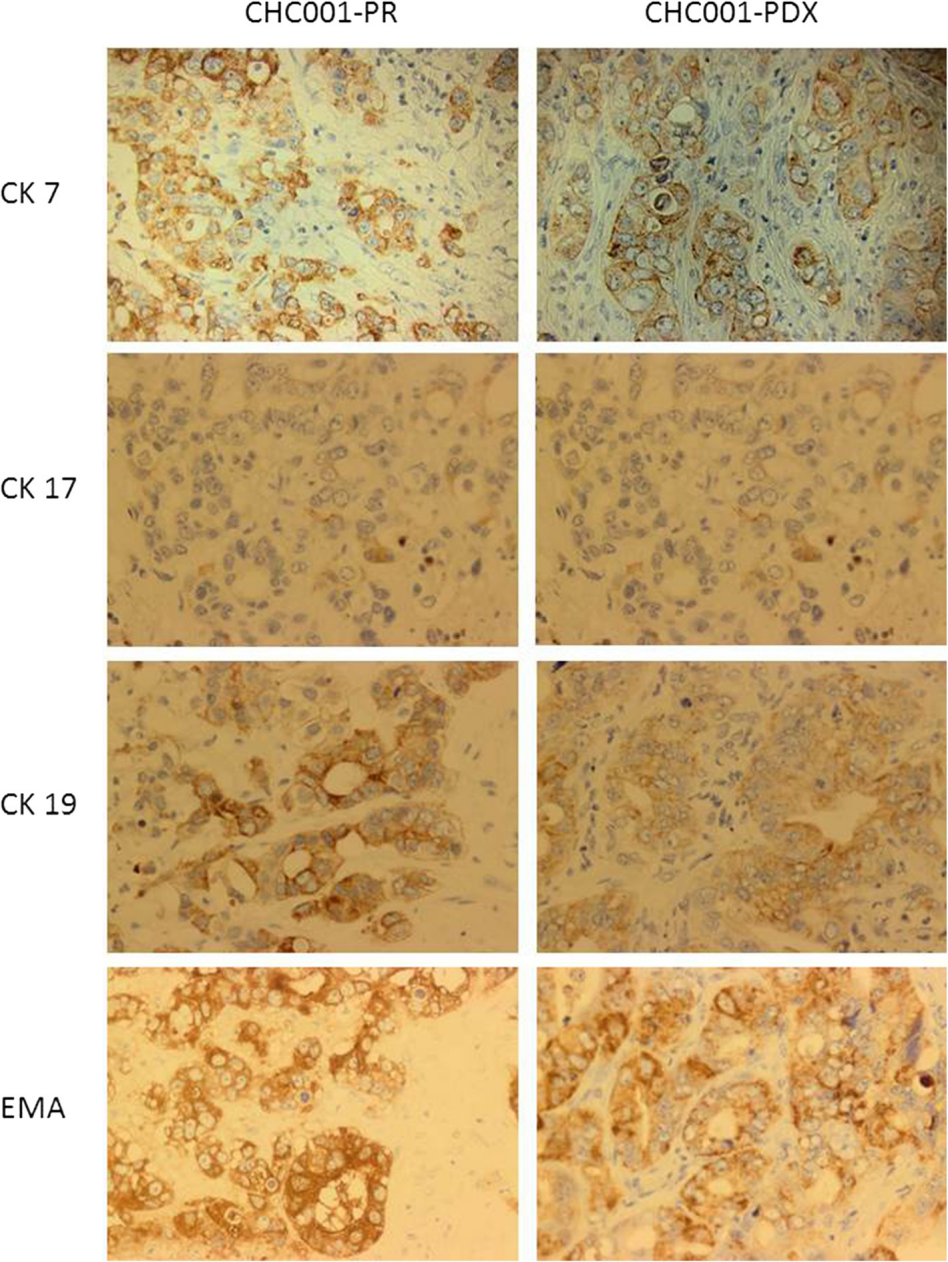
Immunophenotypical tumor features of CHC001 PDX are maintained through serial passages in mice. Cytokeratin 7 (CK7), CK17, CK19 and EMA staining on CHC001-PDX in fourth generation (right panel) was similar to primitive (CHC001-PR) tumor (left panel). Hematoxylin counterstaining, magnification 20X

Immunohistochemistry analysis for the expression of Cytokeratin 7, 17, 19 and EMA as well as the Hematoxillin & Eosin staining [30] showed that PDX retained the same morphology of PR up to the fourth generation as well as the same immunoreactivity (Fig. 1).

### CGH analysis

**Fig. 2:**
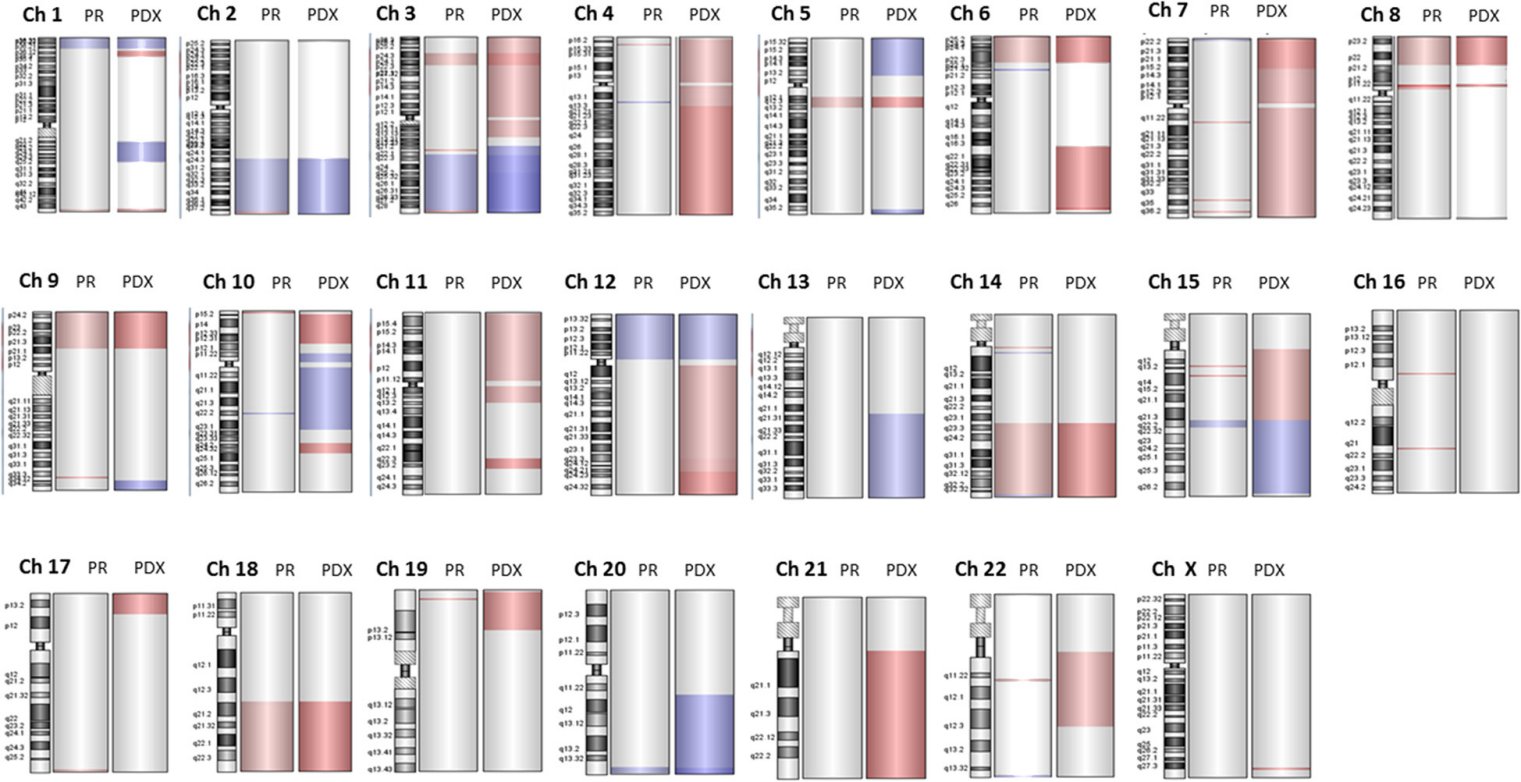
Comparison of chromosomic aberrations in primary tumor (PR) and in its Patient derived xenograft (PDX). In red, the loss regions, in blue the gain regions

The genomic status of PR and of its PDX was assessed by array CGH technique. As shown in Fig. 2, we found a concordance between the two samples (r = 0.64 by Pearson correlation); the number of common chromosomal alteration was 24 with 7 gained regions and 17 lost regions; the most statistically significant chromosome regions included the loss of the regions in 3p, 5q, 6p, 8p, 9p, 14q, 18q, and the gain of the regions 1p, 2q, 3q, and 12p, 15q, and 20q. Table 2 summarizes the common aberrant regions.

**Table 2:** Common aberrant regions between primary and its PDX tumor

GAINED REGIONS				
Chr Name	Start	Stop	Aberration Size	N° of Probes
Chr 1	794595	15755378	14960791	582
chr2	165884672	239972470	74087806	2499
chr3	132814897	195420586	62605690	1960
chr10	76385906	76844343	458439	19
chr12	322142	33190977	32868841	1192
chr15	59862504	63866586	4004083	152
chr20	60606430	62701643	2095216	123
LOST REGIONS				
Chr Name	Start	Stop	Aberration Size	N° of Probes
chr1	246042299	246655416	613118	25
chr3	18852871	32750279	13897409	382
chr4	8003753	8269985	266233	10
chr5	60785924	71460780	10674857	275
chr6	389423	26225376	25835955	858
chr7	73901713	73947170	45458	4
chr7	143425418	143432891	7474	3
chr7	153530377	153586460	56084	3
chr8	176452	22995207	22818758	751
chr8	39237438	39380654	143217	7
chr9	204193	28849141	28644949	888
chr14	63888769	105942876	42054108	1679
chr15	29212452	29253376	40925	4
chr15	34735949	34785082	49134	3
chr18	47738189	78010032	30271844	911
chr19	2936567	3027913	91347	4
chr22	23998465	24040236	41772	4

Further, we revealed that PDX acquired other alterations, in particular the loss of 3p, the entire 4, 6q, the entire 7, 10p, 11p, 12q, 15q, 17p, 19p, 21q and 22q, and the gained regions in 5p, 10q, 13q, 15q, and 20q.

To further characterize the PDX model, we selected genes allocated in the aberrant regions typical of PDX; considering the first 500 amplified or deleted genes, respectively, we performed Gene Ontology (GO) analysis, and GO categories are summarized in Additional file 1: Table S1.

### Gene expression profiling

**Fig. 3:**
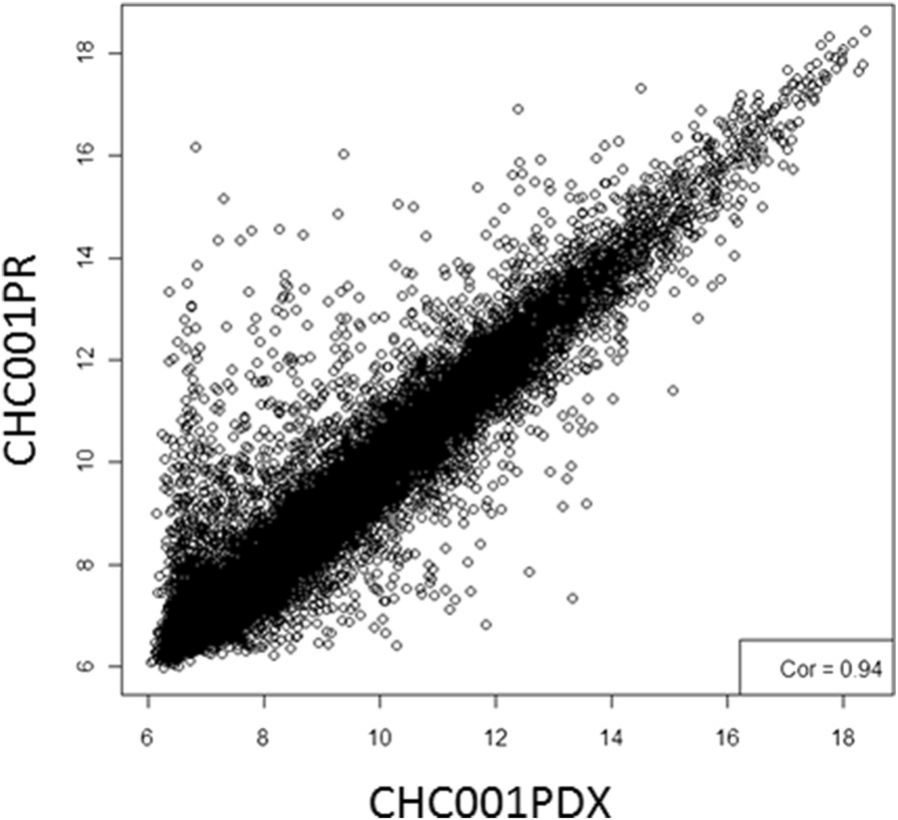
Correlation plot of differentially expressed genes of CHC001PR (primary tumor) and CHC001PDX in fourth generation

Gene expression analysis was performed on the primitive tumor and on the PDX at the fourth passage. Figure 3 showed the correlation plot of differentially expressed genes obtained by Pearson correlation function; this correlation is very high (r = 0.94), enforcing that PDX retained primary tumor characteristics. In order to find the peculiar characteristics of this tumor, common differentially expressed genes were compared to six normal bile duct samples, belonging to the cohort of Andersen and collaborators [14]. Genes list was filtered on adjusted p-value (<0,00001) and the most significant 300 probes were analyzed for Gene Ontology; we found that down-regulated genes are involved in blood coagulation, inflammation response, and in lipid metabolism; on the contrary, up-regulated genes globally affected DNA biosynthesis processes, as nucleosome assembly and organization, translation, underlying that tumor cells are more active rather than normal cells. Even the high correlation of gene expression data, we found 63 up-regulated and 276 down-regulated genes altered in PDX versus primary tumor (Additional file 2: Table S2). Further, we compared differentially expressed genes in PDX with the list of genes allocated in amplified or deleted regions found in PDX; 5 up-regulated and 32 down-regulated genes were found to be overlapped (Additional file 3: Table S3).

### MicroRNA expression profiling

**Fig. 4:**
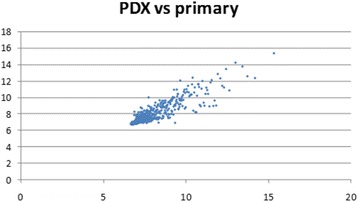
Correlation plot obtained by the microRNA expression values of primary and PDX tumors

**Fig. 5:**
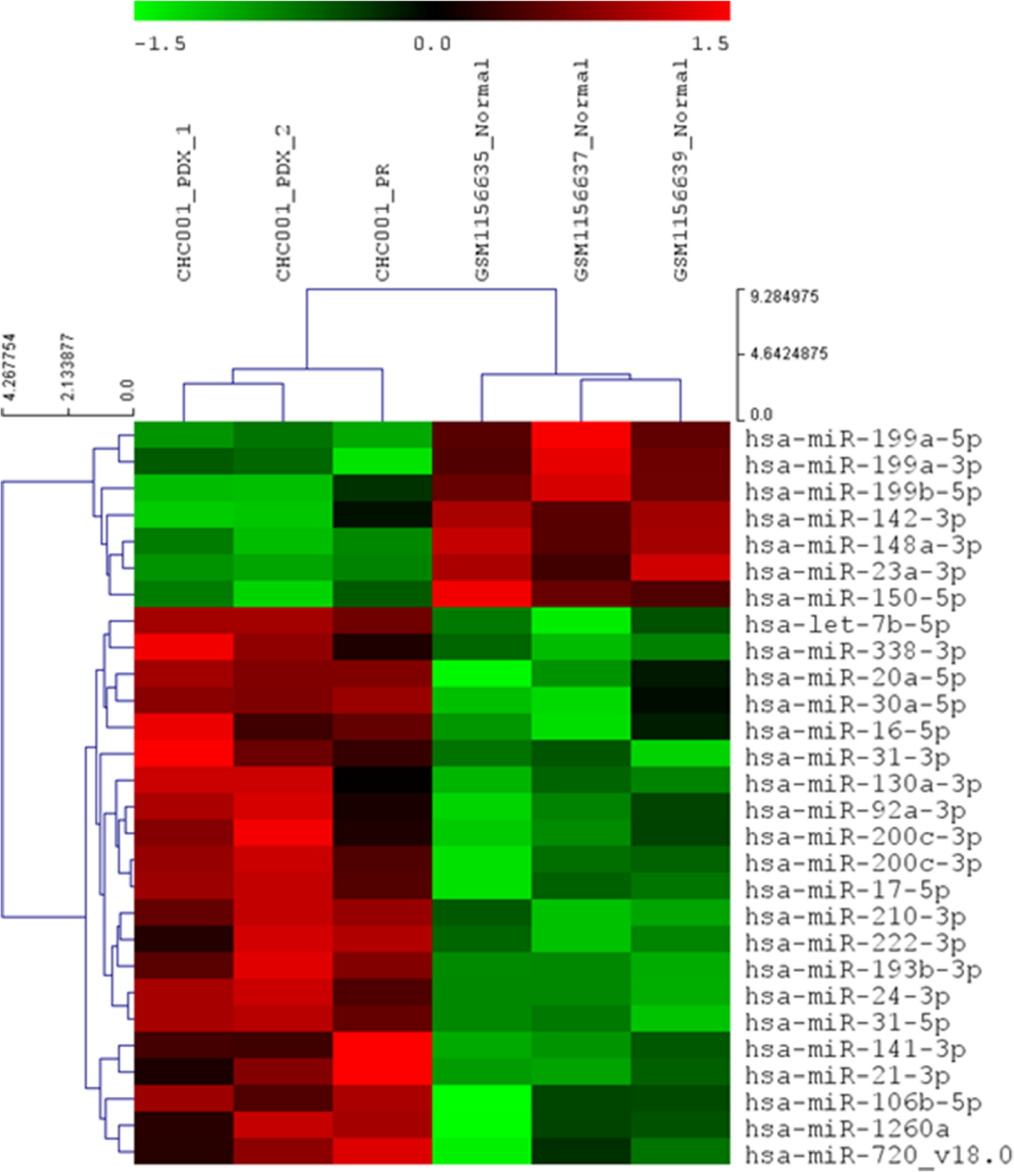
Unsupervised hierarchical cluster showed the different pattern of expression between tumors and normal bile duct

The comparison between PR and its PDX revealed a high correlation in terms of microRNA expression (r = 0.92 by Pearson correlation), as shown in Fig. 4. Common deregulated microRNAs were compared with those obtained by normal bile duct in a work of Peng et al. [31]. Row data were filtered with a logFC < or > 0.58 and a p-value of < 0.01. An unsupervised hierarchical cluster showed the deregulated microRNAs among primary and PDX tumors compared to normal bile duct (Fig. 5). Twenty-eight microRNAs (Table 3), of which 7 down-regulated and 21 up-regulated were selected. Nine out of 28 microRNAs are involved in the negative or positive regulation of cell cycle, apoptosis, migration and proliferation, underlying that these processes are altered in tumor cells. In order to enforce these data, we validated the expression of 4 microRNAs by qRT-PCR. As shown in Additional file 4: Table S4, the trend of expression of mir-21, mir-200, mir-199, and mir-31 is confirmed.

**Table 3:** Common differentially expressed microRNAs obtained by the comparison of tumors (primary and PDX) with normal bile duct

miR name	logFC	P.Value	Function
hsa-miR-142-3p	-1.14661	0.0049146	
hsa-miR-199a-3p	-1.09606	0.0019647	Tumor suppressor
hsa-miR-199a-5p	-0.79526	0.0013074	
hsa-miR-199b-5p	-0.76865	0.0030379	
hsa-miR-148a-3p	-0.75023	0.0008472	
hsa-miR-150-5p	-0.68957	0.000765	Migration/invasion
hsa-miR-23a-3p	-0.66533	0.0002675	
hsa-miR-338-3p	0.767824	0.0037119	Proliferation
hsa-miR-222-3p	0.805174	0.0021683	Proliferation/invasion
hsa-miR-24-3p	0.828359	0.0002201	Proliferation/apoptosis
hsa-miR-92a-3p	0.853479	0.0053925	
hsa-miR-106b-5p	0.878651	0.0057063	
hsa-miR-130a-3p	0.92218	0.0052659	
hsa-miR-16-5p	1.074798	0.0088751	
hsa-miR-31-3p	1.075722	0.0051418	
hsa-miR-20a-5p	1.089514	0.0034313	
hsa-miR-1260a	1.191454	0.0081444	
hsa-miR-193b-3p	1.222449	0.0002997	Tumor suppressor
hsa-miR-21-3p	1.236425	0.0045017	Oncogene
hsa-miR-17-5p	1.326209	0.0009174	
hsa-miR-30a-5p	1.417434	0.0032646	Proliferation/migration
hsa-miR-200c-3p	1.502242	0.0011469	EMT-Transition
hsa-let-7b-5p	1.688168	0.0007466	Proliferation/apoptosis
hsa-miR-210-3p	2.014905	0.0002827	
hsa-miR-31-5p	2.277681	9.30E-05	Cell cycle
hsa-miR-141-3p	2.387609	0.0065583	
hsa-miR-720-v18.0	2.440588	0.0085144	

Furthermore, we analyzed if PDX acquired peculiar characteristics in terms of microRNA expression; Additional file 5: Table S5 showed that only let-7a-5p, miR-15b-5p, let-7d-5p, miR-200b-5p were down-regulated in PDX compared to primary tumor.

### Mutational analysis

**Fig. 6:**
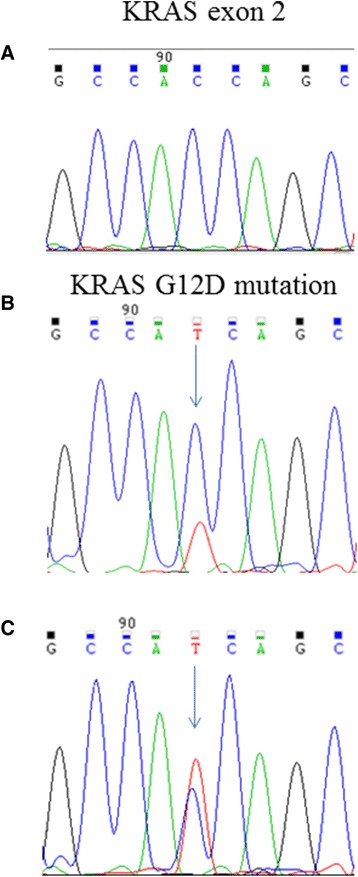
Electropherograms of K-RAS (exon 2). Wilde type sequence of K-RAS exon 2 (**a**), K-RAS G12A mutations found in CHC001 PR (primary tumors) (**b**) and CHC001 PDX in fourth generation (**c**)

Mutational analysis of the kinase domain of EGFR coding sequence, from exons 18 to 21, exons 2,3 and 4 of K-RAS, exons 2,3 and 4 of N-RAS, exons 9 and 20 of PI3KCA, and exon 15 of B-RAF were performed on PR and on PDX. As shown in Fig. 6, only the sequence of K-RAS exon 2 is mutated (G12D mutation) in the primary tumor (panel B) and is maintained in PDX (panel C).

## Discussion

Intrahepatic cholangiocarcinoma constitutes the second most common primary hepatic malignancy with a very poor prognosis [32, 33]. Thus, the identification of alternative therapeutic options is an urgent step to improve the outcome of these patients. ICC PDX models could represent an useful tool either to study the disease from biological and molecular aspects or to investigate response to new therapies. Here, we established and characterized, for the first time, an Italian ICC PDX derived from fresh tumor tissue.

We subcutaneously implanted 17 ICC fresh tumor tissues into immunocompromised mice and we obtained a rate of successful engraftment of 5.8 % (1/17). The engraftment was reached after 4 months from implant, while for the subsequent generations the latency was significantly reduced to one month. The same result was obtained re-implanting archival frozen tissues.

The limited success of engraftment of these tumors is not clear. For colorectal cancer PDX the engraftment rate is 67 % [21]; as concerning mammary tumors, the rate is higher with metastatic tissues rather than primary tumors; moreover, graft achievement depends on other factors, as tumor histotypes, grading, and on the presence of Estrogen and HER2 receptors [34]. We can speculate that the presence of K-RAS mutation in our PDX model could be a driver of the more aggressive phenotype, thus explaining the successful engraftment, as shown in colorectal cancer PDX model [35].

K-RAS mutations are one of the biological determinants of anti-EGFR target therapy resistance in colorectal cancer [36]. Although the role of K-RAS in response to the anti-EGFR therapy in CCA is controversial [37–39], this model could be suitable for the evaluation of the effectiveness of alternative therapies in K-RAS mutated patients for whom anti- EGFR therapies are unfit.

We further compared immunophenotypical and molecular features of PR with its corresponding PDX and we found that both tissue architecture and immunoreactivity of biliary epithelial markers were maintained in PDX.

The genetic relationship between PR and its PDX was established by array-CGH analysis; some genetic alterations were found and maintained from PR to PDX. Some of these regions, in particular the loss of 3p, 6p, 8p, 9p, and 14q regions and the gain of 3q, and 20q regions, are common with the previously found by Miller and coll. in an ICC case series [40]. Other genetic alterations were found only in PDX; this could suggest that, even if the PDX retained the main characteristics of primary tumor, i) the murine environment leads to the acquirement of further chromosomic alterations, ii) the tumor experiences progression regardless of recipient and acquires new chromosomal aberrations, as previously demonstrated by Shiraishi and collaborators [41], iii) the more aggressive cell subpopulation is selected in the murine model.

Comparing transcriptomic profiling of primitive tumor and its PDX, we found a high correlation in terms of gene and microRNA expression, demonstrating that the PDX retained most of primary tumor genetic characteristics. To further characterize our model, we identified a panel of deregulated genes comparing both PR and PDX tumor with published normal bile duct epithelia; to overcome the lack of normal samples in our Institution, we used external dataset of normal biliary tissues, even introducing a possible bias. We select a panel of down-regulated genes involved in blood coagulation, inflammation response, and in lipid metabolism processes and a panel of up-regulated genes involved in DNA biosynthesis processes. We also selected a panel of twenty-eight microRNAs (7 down-regulated and 21 up-regulated), most of them involved in the cell cycle, apoptosis, migration and proliferation regulation. In particular, we found an up-regulation of miR-21, already described in CCA; in fact, the mir-21 overexpression is typical of ICC compared to both normal tissues and hepatic cancer [42, 43]. Moreover, functional studies on CCA cell lines showed the potential oncogenic role of miR-21 by inhibiting PDCD4 and TIMP3, involved in apoptosis and in the inhibition of the matrix metalloproteinases, respectively [44].

## Conclusions

In conclusion, in this study we firstly established an ICC PDX model and characterized it for genetic and molecular alterations; we demonstrated that this model recapitulates the histological characteristics and maintains most of the genetic features of primary tumor, providing a reliable tool to study this neoplasia and to test the efficacy of new drug.

## Additional files

Additional file 1: Table S1. Gene Ontology categories (biological processes, cellular components, molecular functions, and pathways) altered in PDX. (XLSX 15 kb) Additional file 2: Table S2. Differentially expressed genes in PDX versus primary tumor (PR). (XLSX 22 kb) Additional file 3: Table S3. Overlapped deregulated genes obtained comparing differentially expressed genes (GEP) with the list of genes allocated in amplified or deleted regions (aCGH) found in PDX. (XLSX 13 kb) Additional file 4: Table S4. Relative quantitation of deregulated microRNA using the comparative Ct method. (DOCX 18 kb) Additional file 5: Table S5. Deregulated microRNA in PDX compared to primary tumor (PR). (XLSX 9 kb)

## Abbreviations

CCA: Cholangiocarcinoma
ICC: Intrahepatic cholangiocarcinoma
ECC: Extrahepatic cholangiocarcinoma
NGS: Next generation sequencing
PDX: Patient-derived cancer xenograft
CK: Cytokeratin
EMA: Epithelial membrane antigen
IHC: Immunohistochemistry
DAB: 3,3-diaminiobenzidine
aCGH: Comparative genomic hybridization array
FFPE: (Formalin fixed, paraffin embedded)
GEP: Gene expression profiling
DMSO: Dymetil-sulfoxide
FBS: fetal bovine serum
