# Supplementary material for: Establishment of a patient-derived intrahepatic cholangiocarcinoma xenograft model with KRAS mutation
Source: BMC Cancer. 2016 Feb 11;16:90. doi: 10.1186/s12885-016-2136-1 (PMC4750214; doi:10.1186/s12885-016-2136-1)
Supplement: Additional file 4: Table S4. — Relative quantitation of deregulated microRNA using the comparative Ct method. (DOCX 18 kb) [file 12885_2016_2136_MOESM4_ESM.docx]

**Supplementary table 4. Relative quantitation of deregulated microRNA using the comparative Ct method**

| **Sample** | **miR-21** | **RPL21** | **average miR-21** | **average RPL21** | **ΔCt mCtmiR-21-mCtRPL21** | **ΔΔCT** | **2^ΔΔCt** |
| --- | --- | --- | --- | --- | --- | --- | --- |
| **PNLT** | **16.08** | **23.99** | 16.187 | 24.18 | -7.993333333 | 0 | 1 |
|  | **16.27** | **24.28** |  |  |  |  |  |
|  | **16.21** | **24.27** |  |  |  |  |  |
| **PDX** | **14.39** | **22.90** | 14.503 | 22.99 | -8.486666667 | -0.49333 | 1.407694 |
|  | **14.62** | **23.13** |  |  |  |  |  |
|  | **14.50** | **22.94** |  |  |  |  |  |
| **Sample** | **miR-199** | **RPL21** | **average miR-199** | **average RPL21** | **ΔCt mCtmiR-199-mCtRPL21** | **ΔΔCT** | **2^ΔΔCt** |
| **PNLT** | **18.12** | **23.99** | 18.32666667 | 24.18 | -5.853333333 | 0 | 1 |
|  | **18.34** | **24.28** |  |  |  |  |  |
|  | **18.52** | **24.27** |  |  |  |  |  |
| **PDX** | **17.92** | **22.90** | 18.29333333 | 22.99 | -4.696666667 | 1.156667 | 0.448548 |
|  | **18.49** | **23.13** |  |  |  |  |  |
|  | **18.47** | **22.94** |  |  |  |  |  |
| **Sample** | **miR-200** | **RPL21** | **average miR-200** | **average RPL21** | **ΔCt mCtmiR-200-mCtRPL21** | **ΔΔCT** | **2^ΔΔCt** |
| **PNLT** | **24.00** | **23.99** | 24.29 | 24.18 | 0.11 | 0 | 1 |
|  | **24.43** | **24.28** |  |  |  |  |  |
|  | **24.44** | **24.27** |  |  |  |  |  |
| **PDX** | **14.95** | **22.90** | 15.09666667 | 22.99 | -7.893333333 | -8.00333 | 256.5922 |
|  | **15.23** | **23.13** |  |  |  |  |  |
|  | **15.11** | **22.94** |  |  |  |  |  |
| **Sample** | **miR-31** | **RPL21** | **average miR-31** | **average RPL21** | **ΔCt mCtmiR-31-mCtRPL21** | **ΔΔCT** | **2^ΔΔCt** |
| **PNLT** | **26.79** | **23.99** | 26.73 | 24.18 | 2.55 | 0 | 1 |
|  | **26.72** | **24.28** |  |  |  |  |  |
|  | **26.68** | **24.27** |  |  |  |  |  |
| **PDX** | **16.57** | **22.90** | 16.64666667 | 22.99 | -6.343333333 | -8.89333 | 475.5105 |
|  | **16.57** | **23.13** |  |  |  |  |  |
|  | **16.80** | **22.94** |  |  |  |  |  |

The ΔCt value is determined by subtracting the average RPL21 Ct value from the average selected microRNA Ct values.

The calculation of ΔΔCt involves subtraction by the ΔCt calibrator value (pool of normal liver tissue: PNLT).

The range given for selected microRNA relative to calibrator value is determined by evaluating the expression 2^- ΔΔCt^
